# Diseases of the Rectum and Anus

**Published:** 1894-02

**Authors:** 


DISEASES OF THE RECTUM AND ANUS, THEIR PATHOLOGY, DIAGNOSIS, AND TREATMENT. By Charles B. Kelsey, A. M., M. D., New York, Professor of Diseases of the Rectum, at the New York Post-Graduate Medical School and Hospital; late Professor of Diseases of the Rectum at the University of Vermont, etc. Fourth edition, Revised and Enlarged. With two Chromo-Lithographs and one hundred and sixty-two Illustrations. Octavo, 496 pages, extra muslin, price $4. New York: William Wood & Company.

This very popular work has now reached its fourth edition, and continues to grow in professional favor. As a teacher in one of the largest clinical schools in this country Dr. Kelsey has had ample facilities for studying the different diseases of the rectum and anus, and he he is now considered one of the standard authorities on this specialty. The present edition is fully up to the times, eminently practical, and should be in the library of every general practitioner as well as specialist.

The author devotes more than fifty pages to the consideration of hemorrhoids, giving the different varietes and the several modes of treatment now in vogue. He was formerly a strong advocate of the injection method of treatment and was first driven to using it by quacks and pretenders. After a more thorough trial and a longer experience, he, like Dr. Andrews, of Chicago, has abandoned this treatment except in a few selected cases. His own preference is for the clamp and cautery in the majority of cases, though he sometimes uses the ligature or the carbolic acid injection.

Each chapter in this book will be of much interest and worth to the profession generally, but we believe that the one on hemorrhoids will prove of more benefit to the general practitioner than any of the others, and it alone is more than worth the price of the book. H.

